# Red blood cell distribution width: Genetic evidence for aging pathways in 116,666 volunteers

**DOI:** 10.1371/journal.pone.0185083

**Published:** 2017-09-28

**Authors:** Luke C. Pilling, Janice L. Atkins, Michael O. Duff, Robin N. Beaumont, Samuel E. Jones, Jessica Tyrrell, Chia-Ling Kuo, Katherine S. Ruth, Marcus A. Tuke, Hanieh Yaghootkar, Andrew R. Wood, Anna Murray, Michael N. Weedon, Lorna W. Harries, George A. Kuchel, Luigi Ferrucci, Timothy M. Frayling, David Melzer

**Affiliations:** 1 Epidemiology and Public Health Group, University of Exeter Medical School, RILD Level 3, Royal Devon & Exeter Hospital, Exeter, EX2 5DW, United Kingdom; 2 Department of Genetics and Genome Sciences, Institute for Systems Genomics, University of Connecticut Health Center, Farmington, Connecticut, United States of America; 3 Genetics of Complex Traits Group, University of Exeter Medical School, RILD Level 3, Royal Devon & Exeter Hospital, Exeter, EX2 5DW, United Kingdom; 4 Department of Community Medicine and Health Care, Connecticut Institute for Clinical and Translational Science, Institute for Systems Genomics, University of Connecticut Health Center, Farmington, Connecticut, United States of America; 5 Institute of Biomedical and Clinical Sciences, University of Exeter Medical School, RILD Level 3, Royal Devon & Exeter Hospital, Exeter, United Kingdom; 6 Center on Aging, University of Connecticut, Farmington, CT, United States of America; 7 National Institute on Aging, Baltimore, MD, United States; Johns Hopkins University, UNITED STATES

## Abstract

**Introduction:**

Variability in red blood cell volumes (distribution width, RDW) increases with age and is strongly predictive of mortality, incident coronary heart disease and cancer. We investigated inherited genetic variation associated with RDW in 116,666 UK Biobank human volunteers.

**Results:**

A large proportion RDW is explained by genetic variants (29%), especially in the older group (60+ year olds, 33.8%, <50 year olds, 28.4%). RDW was associated with 194 independent genetic signals; 71 are known for conditions including autoimmune disease, certain cancers, BMI, Alzheimer’s disease, longevity, age at menopause, bone density, myositis, Parkinson’s disease, and age-related macular degeneration. Exclusion of anemic participants did not affect the overall findings. Pathways analysis showed enrichment for telomere maintenance, ribosomal RNA, and apoptosis. The majority of RDW-associated signals were intronic (119 of 194), including SNP rs6602909 located in an intron of oncogene *GAS6*, an eQTL in whole blood.

**Conclusions:**

Although increased RDW is predictive of cardiovascular outcomes, this was not explained by known CVD or related lipid genetic risks, and a RDW genetic score was not predictive of incident disease. The predictive value of RDW for a range of negative health outcomes may in part be due to variants influencing fundamental pathways of aging.

## Introduction

Increased variation in a person’s Red Blood Cell (RBC) volumes (RBC distribution width (RDW), also termed anisocytosis) is strongly predictive of a range of incident cardiovascular conditions, cancers and mortality [1–3]. Although RDW is routinely measured in clinical hematology reporting–it is calculated by dividing the standard deviation of mean cell volume (MCV) by the MCV and multiplying by 100, to yield a RDW percentage [4]–it is only used clinically for diagnosis of anemia subtypes. Understanding the mechanisms involved in the links between increased RDW and negative health outcomes could provide clues to potential interventions to improve prognosis in those with high RDW who are not anemic, particularly in older people.

Established clinical causes of increased RDW include anemia and other iron or folate deficiencies [5], dyslipidemia [6] and other metabolic abnormalities, and inflammation [7]. Proposed mechanisms for increased RDW also include impaired erythropoiesis (the generation of new RBC) perhaps due to effects of inflammation or senescence of erythropoietic cells in the bone marrow, plus variations in RBC survival [8]. A previous analysis of 36 blood cell traits identified genetic variants [9], but the genetic signals for RDW were not investigated in depth in relation to the mechanisms that might explain the predictive value of RDW for negative health outcomes in people with normal hemoglobin levels.

We aimed to investigate RDW (overall and excluding anemia) using genetic analysis in a large population cohort, to identify underlying mechanisms. This involved genome-wide analysis of associations to find independent signals, investigations of biological pathways implicated by the results, and overlap with known risk alleles. We also examined associations between RDW and known variant genetic risk score analysis for conditions predicted by RDW, including cardiovascular disease. For this analysis, we used the exceptionally large UK Biobank volunteer sample with standardized measures of RDW across the cohort.

## Results

We included 116,666 UK Biobank participants of white/British descent with complete hematology measures, covariate data, and genetics data from the interim data release (May 2015) in our analyses. The mean age was 57 years (SD: 7.9, min = 40, max = 70), with 52,541 aged 60 to 70 years old, and the majority (52.6%) were female: **Table 1**.

**Table 1:** Summary statistics for 116,666 UK Biobank participants.

Trait		Mean (SD)	Min—Max
Age (years)		56.92 (7.94)	40–70
RDW (%)		13.49 (0.95)	11.1–38.3
Mean Cell Volume (fL)		91.39 (4.45)	54.5–160.3
Hemoglobin conc. (g/dL)		14.23 (1.23)	0.14–20.5
Sex		**N**	**%**
	*Females*	61,306	52.55
	*Males*	55,361	47.45
Anemia			
	*No*	98,871	84.75
	*Yes* *	17,795	15.25
RDW (%)			
	*<12*.*5*	8,703	7.46
	*12*.*5–12*.*9*	24,559	21.05
	*13*.*0–13*.*4*	33,804	28.97
	*13*.*5–13*.*9*	25,413	21.78
	*14*.*0–14*.*4*	12,967	11.11
	*14*.*5–14*.*9*	5,460	4.68
	≥*15*.*0*	5,761	4.94

* = either hospital diagnosis or raised hemoglobin (WHO definition, see methods)

### Variance explained by genotypes

We estimated that 29.3% (SE = 0.5%) of the variance in RDW was accounted for by 457,643 directly genotyped variants with MAF>0.1%. In a secondary analysis we estimated the proportion explained in two groups: those aged ≥60 years (n = 52,541), and those aged <50 years (n = 24,988). The proportion of variance in RDW explained by the genetic variants was greater in the older group (33.8%, SE = 1.0%) compared to the younger group (28.4%, SE = 2.0%), and this difference is statistically significant (p = 0.012).

In sensitivity analyses including only those directly genotyped SNPs associated with RDW (at 2 separate p-value cut-offs: p<5x10^-5^ and p<5x10^-8^; n SNPs = 4,110 and 2,095 respectively) we observed a reduction in the variance in RDW explained, as expected, but the conclusions remained consistent: in all participants the variance in RDW explained by the included SNPs was 18% (SE = 0.6%) and 14.2% (SE = 0.7%) for the 2 p-value thresholds respectively. There was still a marked difference in the variance explained between the older group (19.6%, SE = 0.8%; and 15.4%, SE = 0.8%) and the younger group (17.3%, SE = 0.9%; and 13.5%, SE = 1%).

We estimated the proportion of variance in CHD (10,280 cases in 116,666 participants) accounted for by the variants to be 5.95% (SE = 0.45%). The proportion of the variance shared between RDW and CHD attributable to genetics is 6.62% (SE = 2.69%: 95% CIs = 1.35 to 11.9%).

### Genome-wide association study

**Fig 1:**
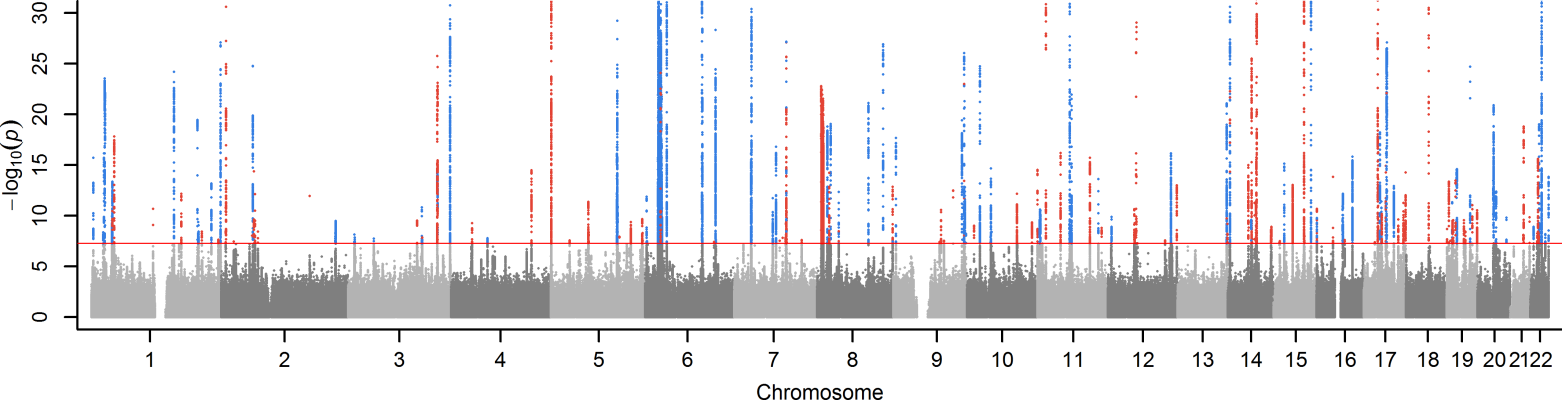
Genetic variants associated with RDW in GWAS of 116,666 UK Biobank participants. The variants are grouped into 194 independent signals, colored blue if a variant in the signal is associated with any trait in the NHGRI-EBI GWAS catalog of known associations, otherwise colored in red. The y-axis (–log_10_ *p*-values) is limited to 30 for clarity, as the max value is 200. See **S1 Table** for RDW associations for each signal, and **S4 Table** for mapping to the catalog. Horizontal line *p* = 5x10^-8^.

Of the 16,832,071 genetic variants included in this GWAS, 30,988 were significantly (p<5x10^-8^) associated with RDW (**Fig 1**; full results available to download here: https://doi.org/10.6084/m9.figshare.5395504.v1) after adjustment for age, sex, assessment center and array type (genetic relatedness is accounted for in the linear mixed models approach so no PCs are included–see methods). A quantile-quantile plot shows clear inflation (**S1 Fig**; Lambda = 1.15), consistent with polygenic inheritance of many causal variants of small effects which can now be discovered using large sample sizes [10]. The 30,988 variants were mapped to 141 loci (runs of variants separated by <2Mb) on the genome, and included 194 independent signals after conditional analysis (“conditional SNPS”) (**S1 Table**).

After excluding 17,795 participants with anemia the GWAS of RDW was repeated on the 98,871 remaining participants; all 194 conditional SNPs remained nominally associated with RDW (p<0.001), but 24 were no longer genome-wide significant (p<5x10^-8^), possibly due to reduced power in the smaller sample size. All subsequent analyses are based on the results from the analysis of all participants.

### Functional implications of RDW-associated genetic variants

We utilized the UCSC Variant Annotation Integrator (http://www.genome.ucsc.edu/cgi-bin/hgVai) and the Ensembl Variant Effect Predictor (http://grch37.ensembl.org/Homo_sapiens/Tools/VEP) to interrogate a number of genomic annotation databases for the conditional SNPs. The majority (119 out of 194 total) were intronic, and 12 were located in the 3’ or 5’ un-translated regions (see **S2 Table** for complete variant annotation output). We found that 37 of 194 independent RDW-associated signals are known eQTLs (i.e. affect the expression of a gene) in whole blood (http://genenetwork.nl/bloodeqtlbrowser): 15 of these affect the gene predicted by the variant annotation integrator, including rs6602909, located in an intron of oncogene *GAS6* [11] (see **S3 Table** for complete matching of RDW-associated signals to eQTL data). SNP rs7775698 is an trans-eQTL for more than 30 genes.

Fifteen of the RDW-associated signals were exonic: 11 missense, 3 synonymous, and a 17 base-pair exonic deletion in gene *SMIM1*. The missense and deletion variants are shown in **Table 2**. PolyPhen-2 predicted that three missense variants are “probably damaging” to the protein function of genes *TRIM58*, *PLD1* and *PNPLA3*. Variant rs2075995 was predicted to be “possibly damaging” to the protein function of gene *E2F2*. *TRIM58* is a ubiquitin ligase induced during late erythropoiesis [11]; *PLD1* is a phospholipase implicated in processes including membrane trafficking [11]; *PNPLA3* is a triacylglycerol lipase in adipocytes and variant rs738409 is associated with susceptibility to Non-alcoholic Fatty Liver Disease [12]. Gene *SMIM1* is involved in RBC formation [11] and a 17 base-pair deletion (rs566629828) causing an exonic frameshift is strongly associated with increased RDW.

**Table 2:** Four RDW-associated conditional genetic variants may have damaging effects on proteins.

		Position								RDW association		
Variant		CHR: POS	cDNA	Protein	eA	Gene	AA	Codon	Effect	Beta	P	eAF
**SNP** *												
	rs2075995	1:23847464	1105	226	A	*E2F2*	Q/H	caG/caT	P	-0.033	1.8x10^-16^	0.502
	**rs3811444**	1:248039451	1169	374	T	*TRIM58*	T/M	aCg/aTg	**D**	-0.066	6.3x10^-59^	0.333
	rs143845082	3:171417570	1539	398	A	*PLD1*	R/C	Cgt/Tgt	P	0.217	4.6x10^-13^	0.005
	**rs149535568**	3:171442535	1056	237	A	*PLD1*	G/C	Ggc/Tgc	**D**	0.27	9.3x10^-15^	0.048
	rs10479001	5:131607721	751	225	T	*PDLIM4*	A/V	gCa/gTa	B	0.056	1.1x10^-8^	0.042
	rs2578377	5:153413390	459	122	T	*FAM114A2*	G/S	Ggt/Agt	B	0.026	4.1x10^-10^	0.633
	rs1799945	6:26091179	347	63	G	*HFE*	H/D	Cat/Gat	B	-0.138	2.2x10^-143^	0.150
	rs368865	13:113479820	1037	317	G	*ATP11A*	M/V	Atg/Gtg	B	0.025	2.4x10^-9^	0.724
	rs556052	19:49377436	1215	316	C	*PPP1R15A*	A/P	Gct/Cct	B	-0.028	8.5x10^-12^	0.333
	rs855791	22:37462936	2321	736	T	*TMPRSS6*	V/D	gTc/gAc	B	0.119	1.2x10^-204^	0.439
	**rs738409**	22:44324727	617	148	G	*PNPLA3*	I/M	atC/atG	**D**	-0.029	6.2x10^-9^	0.216
**Deletion ¥**												
	**rs566629828**	1:3691997–3692014	309–325	21–26	Del	*SMIM1*	-	-	**F**	0.12	4.5x10^-12^	0.013

* Output from UCSC Variant Annotation Integrator for the RDW-associated conditionally independent SNPs located in protein-coding regions.

¥ Output from Ensembl Variant Effect Predictor for insertion/deletion events.

“Effect” = (D)amaging, (P)ossibly damaing, or (B)enign SNP, or (F)rameshift due to deletion; “AA” = amino-acid change; “eA” = effect allele (positive strand) causing the change; “Beta” = the beta coefficient for the effect allele on RDW; "P" = p-value for the RDW association; “eAF” = effect allele frequency in UK Biobank white/British participants. All positions are from hg19/b37.

### GWAS catalogue of known genetic associations

Of the 194 conditional SNPs associated with RDW, 77 mapped to at least one trait in the catalogue of published GWAS (downloaded 13^th^ March 2017). This was arrived at by; filtering the 33,005 SNP-trait associations to those with p<5x10^-8^ (leaving 14,148 SNP-trait pairs for analysis); matching the positions to the UK Biobank results (13,146); filtering to those with significant RDW association in our analysis (p<5x10^-8^), leaving 923 SNP-trait pairs (420 unique SNPs; some SNPs are associated with multiple traits). These 420 unique SNPs mapped to 77 of the 194 conditional SNPs associated with RDW. These are shown in **Fig 1**; see **S4 Table** for further detail.

Traits also associated with the individual RDW variants included iron metabolism and several other red cell measures. Variants present were also associated with BMI, several lipids, hemoglobin A1C and metabolic syndrome, as well as height. Autoimmune associated conditions included autoimmune thyroid disease, type 1 diabetes, Crohn’s disease, inflammatory bowel disease, rheumatoid arthritis, systemic lupus erythematosus and ulcerative colitis. Variants linked to Lung, ovary and nasopharyngeal cancers were present. In addition, conditions associated with aging were represented, including variants linked to Alzheimer’s disease, age at menopause, bone density, Myositis, Parkinson’s disease, macular degeneration, C-reactive protein levels and longevity. For Alzheimer’s and longevity these were known SNPs in the *APOE* gene region.

### Gene ontology enrichment

MAGENTA software [13] identified pathways enriched in the genes mapped to variants significantly associated with RDW, including telomere maintenance, ribosomal RNA transcription and histone modifications (**Table 3**), plus apoptosis. In addition, pathways related to lipid metabolism (in particular chylomicrons) were also enriched. Additional information, such as the expected/observed number of associated genes for each pathway is included in **S5 Table**.

**Table 3:** MAGENTA results: Biological pathways enriched in RDW genetics signals.

Biological pathway		N genes	Exp. Sig.	Obs. Sig.	p-value
*Chromosomal & DNA-related*					
	Histone	33	2	13	9.9x10^-7^
	Nucleosome	31	2	9	1.7x10^-5^
	Nucleosome assembly	43	2	10	1.0x10^-4^
	Packaging of telomere ends	23	1	11	9.9x10^-7^
	Telomere maintenance	49	2	12	5.0x10^-6^
	Chromatin packaging and remodeling	142	7	20	2.4x10^-5^
	Apoptosis-induced DNA fragmentation	9	0	4	4.0x10^-4^
*RNA polymerase-related*					
	RNA Pol I—promoter clearance	44	2	14	9.9x10^-7^
	RNA Pol I—promoter opening	23	1	14	9.9x10^-7^
	RNA Pol I—chain elongation	30	2	9	7.0x10^-6^
	RNA Pol I, III, and mitochondrial transcription	82	4	15	1.2x10^-5^
*Lipid-related*					
	Chylomicron	9	0	5	3.4x10^-5^
	Chylomicron-mediated lipid transport	15	1	5	6.0x10^-4^
	Phospholipid efflux	8	0	4	2.0x10^-4^
	Very-low-density lipoprotein particle	14	1	5	2.0x10^-4^
*Other*					
	Systemic Lupus Erythematosus	60	3	15	9.9x10^-7^
	Olfaction	105	5	18	4.0x10^-6^
	Cellular iron ion homeostasis	25	1	8	2.8x10^-5^
	Lactation (mammary development)	8	0	3	4.5x10^-3^
					

Output from MAGENTA GWAS enrichment software for the 30,988 RDW-associated genetic variants. Pathways shown are those where the estimated false discovery rate p<0.05. N genes = number of genes in the gene-set analysed; Exp. Sig. = Expected number of genes with a corrected gene p-value above the 95 percentile enrichment cutoff; Obs. Sig. = Observed number of genes with a corrected gene p-value above the 95 percentile enrichment cutoff; p-value = nominal p-value using 95 percentile of all gene scores for the enrichment cutoff.

### Genetic risk score associations

**Fig 2:**
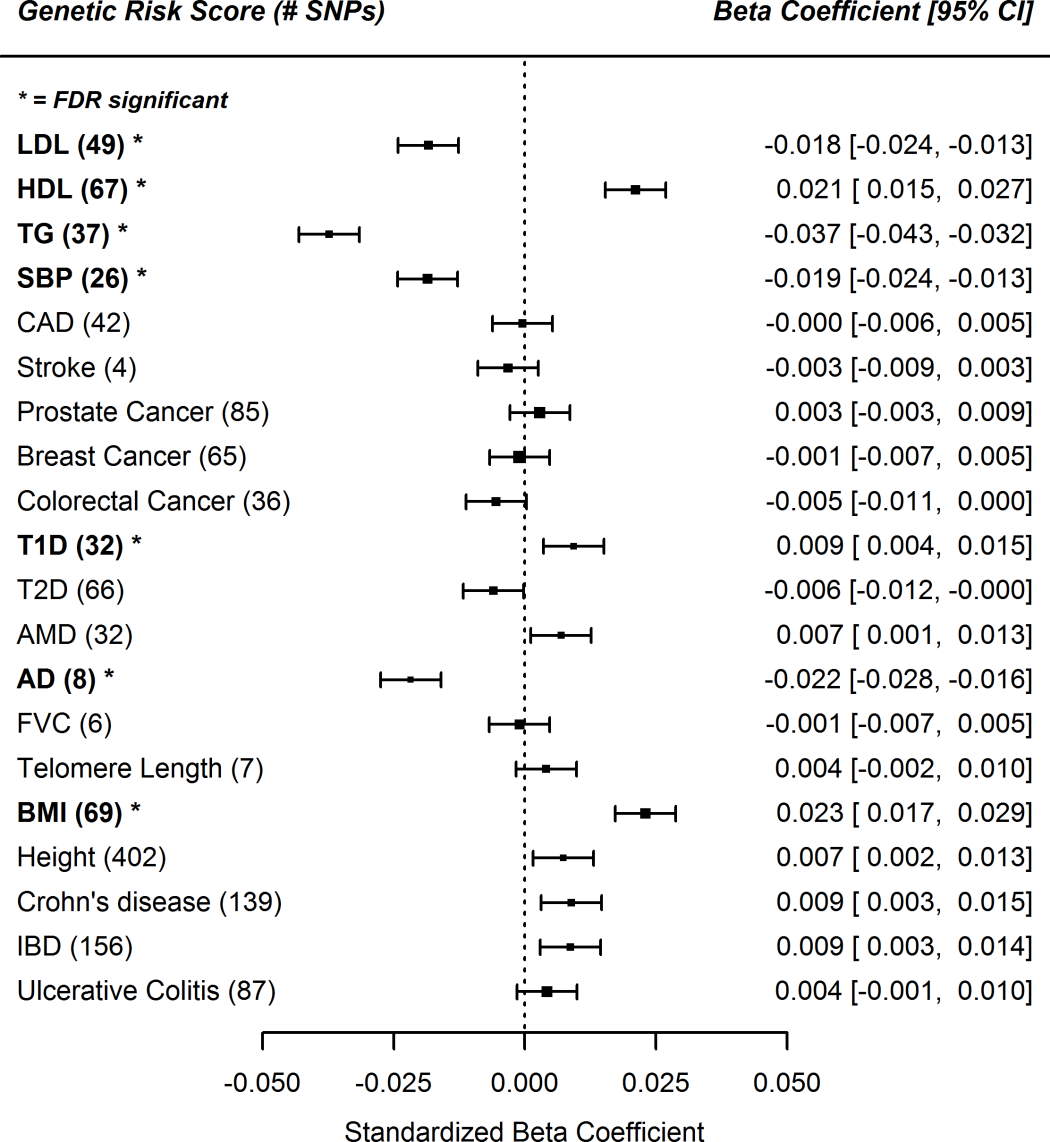
Genetic Risk Score associations with RDW. * FDR = false-discovery rate adjusted significant association. Genetic Risk Scores (GRS) were z-transformed prior to analysis. Linear regression model against RDW (z-transformed) including 116,666 participants, adjusted for age, sex, assessment center and population structure (genetic PCs 1–5). LDL (low-density lipoprotein), HDL (high-density lipoprotein), TG (triglycerides), SBP (systolic blood pressure), CAD (coronary artery disease), T1D (type-1 diabetes), T2D (type-2 diabetes), AMD (age-related macular degeneration), AD (Alzheimer’s disease), FVC (forced vital capacity), BMI (body mass index), IBD (inflammatory bowel disease). Full results in **S6 Table**.

We tested 20 Genetic Risk Scores (GRS) for potentially explanatory traits for the predictive value of RDW for negative health outcomes; 7 were significant after adjustment for multiple testing (p<0.0025). Three were associated with raised RDW (HDL, type-1 diabetes, and BMI); four were associated with lower RDW (triglycerides, LDL, systolic blood pressure, and Alzheimer’s disease) (**Fig 2; S6 Table**). After exclusion of the ApoE locus the HDL GRS remained significant (p = 0.003), but the AD GRS was no longer significant (p = 0.84).

Genetic risks for Crohn’s disease and inflammatory bowel disease were nominally associated with increased RDW (Beta = 0.009: 95% CIs = 0.003 to 0.015; Beta = 0.009: 95% CIs = 0.003 to 0.014; respectively). Risk scores for coronary artery disease were not associated with RDW, including after removal of the lipid related variants (Beta = 0.00: 95% CIs = -0.006 to 0.005). A genetic risk score for telomere length was not significantly associated with RDW.

We also created a GRS for RDW using the 194 conditional SNPs identified in this analysis. Although the GRS was associated with anemia (OR = 1.10: 95% CIs = 1.08 to 1.12) there was no association with mortality, coronary artery disease, or cancer (p = 0.24, p = 0.24, and p = 0.44, respectively). In sensitivity analyses excluding RDW-SNPs associated with CAD p<5x10^-8^ in a recent meta-analysis [14], in case results were biased due to invalid genetic instruments, or using the MR-Egger method to check for pleiotropy [15], the conclusions remained consistent.

## Discussion

Variation in RBC size (RBC Distribution Width, RDW) increases markedly with age [16] and high RDW values are strongly predictive of increased mortality, plus incident cardiovascular disease and certain cancers [3,17]. However, RDW is not generally considered as being a clinically useful measure outside the assessment of anemia sub-type, perhaps because the mechanisms explaining its prognostic value in people without anemia are unclear. In this study we investigated RDW using genetic analysis to understand the molecular mechanisms underpinning variation in RBC size.

A large proportion of RDW variation (29.3%) was attributable to common genetic variants in this analysis, and the variation explained by genetic variants appeared to increase with age, contrary to common assumptions that genetic effects decrease with advancing age. This could be due to effects accumulating over a time. Many of the “hallmarks of aging” also have this property [18]. Additionally, many of the RDW-associated genetic variants (in 71 of 194 conditionally independent signals) have previously been associated with other traits including metabolic syndrome, certain cancers, and autoimmune disease as well as aging related conditions including menopausal age.

Our analysis of genetic risk scores (GRS) showed that participants with genetically-increased risk for coronary artery disease or cancer did not have significantly higher RDW. Similarly, we found that a GRS for RDW (using the 194 variants) had was not associated with mortality, incident coronary heart disease (CHD), or cancer. Consistent with this, the proportion of the variance shared between RDW and CHD attributable to genetics was only 6.6%; therefore the majority of genetically influenced red cell variation is independent of CHD.

As higher RDW is associated with CVD epidemiologically, we might have hypothesized that genetic risks for adverse lipid levels or blood pressures affect RDW, but instead we found associations in the opposite directions: GRS analysis showed that participants with genetically lower LDL levels, triglyceride levels, or systolic blood pressure, had higher RDW, and genetically higher HDL was associated with greater RDW. Published observational associations between lipids and RDW are only partially consistent with our findings here: Lippi *et al* found that LDL and HDL cholesterol were both negatively related to RDW, and triglyceride levels were positively related [6]. It is known that RBC have a role in cholesterol homeostasis by transporting cholesterol in the plasma membranes, with significant inter-individual differences not entirely explained by age or cholesterol levels [19]. The relationship between lipids and RDW is complex and requires further investigation. We also observed that genetically increased risk of type-1 diabetes was associated with increased RDW–further evidence for autoimmune involvement, in addition to the overlap in significant SNPs in the GWAS catalogue–and that genetically increased BMI is associated with increased RDW.

Genetic variants associated with RDW were enriched in expected pathways, including iron homeostasis, but we also found evidence for telomere maintenance, ribosomal RNA production, and a number of nucleosome and histone pathways. Short telomere length is a hallmark of cellular aging to senescence [18] and is associated with many risk factors of disease, however the causal direction is still uncertain [20], and longer telomeres have been linked to risk of cancer [21]. Kozlitina *et al* reported in 2012 that increased RDW is associated with shorter telomeres in leukocytes [22]. We created a genetic risk score for telomere length but it was not associated with RDW (Beta = 0.004: 95% CIs = -0.002 to 0.010); further work is required to clarify this association.

We also found enrichment of RNA polymerase I (which transcribes ribosomal RNA) and RNA polymerase III (which transcribes transfer RNA); both are required for protein synthesis, including hemoglobin, and can even function as regulators of gene expression in their own right [23], suggesting these are key factors for consistent production of RBC. Deregulation of transcription and proteostasis are hallmarks of aging [18], and we have previously reported deregulation of gene expression of the transcriptional machinery with advancing age [24].

Four of the conditionally independent genetic variants associated with RDW are exonic and affect the amino-acid sequence of the protein products. Most others are intronic or intergenic, and may be regulatory; this is supported by published eQTL data [25], in which 37 of the RDW-associated signals have been reported to affect the expression of a gene in whole blood. Of the 194 independent variants identified, 119 are intronic; this does not appear to be an uncommonly high proportion, as 23 of 39 novel BMI variants identified by Locke *et al*. 2015 were intronic [26] and 6 of 12 novel blood pressure variants identified by Kato *et al*. 2015 were intronic [27]. Intronic variants may have direct effects on protein expression by affecting the splicing or processing of the pre-mRNA, a complex process to produce the correct mRNA and subsequent protein [28], or by altering expression of a regulatory non-coding RNA such as a micro RNA. These will be useful targets for future research to determine how these variants ultimately affect consistency in red blood cell size.

### Limitations

The UK Biobank is a volunteer study which achieved only a 5% response rate, so at assessment the participants were healthier than the general population. However, there was substantial variation in RDW within the participants so the results can still be generalized to the wider population [29].

More work is needed to replicate these findings in independent cohorts, and to establish the effects in other populations, particularly non-European ancestries. No data have been released regarding the UK Biobank participant’s lipid and other blood assays; once this is available, further investigations into the complex relationship between RDW and lipids can be performed.

The GWAS catalogue does not contain every published GWAS, especially the most recent studies, but contains many of the largest meta-analyses for traits such as cardiovascular disease and cancer. It is therefore likely we have missed some studies using this method, therefore our results present an approximation of the overlap between RDW signals and other traits.

### Conclusions

Variation in RDW has a substantial genetic component, and this increases with increasing age. Although increased RDW is predictive of cardiovascular outcomes, this was not explained by known CVD or related lipid genetic risks. The predictive value of RDW for a range of negative health outcomes may in part be due to variants influencing fundamental pathways of aging, but it may also be reflective of exposures or underlying conditions.

## Materials and methods

The UK Biobank study recruited 503,325 volunteers aged 40–70 who were seen between 2006 and 2010. Data includes RBC distribution width (RDW) and other clinical hematology measures, extensive questionnaires including smoking behavior and education history, and follow-up using electronic medical records. Currently one third of the participants have available genotype information. We utilized data from 116,666 participants of white/British descent with all available data. UK Biobank has approval from the North West Multi-centre Research Ethics Committee (MREC), which covers the UK. UK Biobank data is available to all bona fide researchers, in the UK and internationally, without preferential access. Applications are reviewed, with ethical advice from from both the University of Oxford’s Ethox Centre and the Ethics and Governance Council, where appropriate. Following approval and signing of a materials transfer agreement, de-identified data is transferred to researchers [30]. Information and access to data can be found online (www.ukbiobank.ac.uk/scientists).

### Phenotypes

RDW is a measure of the variability in the mean size of the RBC in each participant (in % units). It was measured using four Beckman Coulter LH750 instruments within 24 hours of blood draw, with extensive quality control performed by UK Biobank [31]. RDW is a continuous, highly skewed trait, therefore we used quantile normalization of the continuous measure to create a Gaussian distribution, so that the normality assumption of the linear regression models were not violated.

Anemia was determined both using self-reported diagnosis, electronic medical records (ICD10: D64* and D5* categories), or by low hemoglobin levels at the baseline assessment (<120g/L in females, <130g/L in males: from WHO definition [32]).

Coronary heart disease (CHD) was defined using self-reported myocardial infarction or angina, or diagnosis in the electronic medical records (ICD10: I20-I25).

### Variance explained and genetic correlation

Variance-components analysis determines the heritable phenotypic variation in complex traits explained by genetic variants. We used BOLT-REML to determine the variance in RDW explained by the common, genotyped variants (n = 457,643 directly genotyped variants with minor allele frequency (MAF) >0.1%, HWE p>1x10^-6^ and missingness <1.5%) using restricted maximum likelihood estimation [33]. The method performs multiple iterations to approximate BOLT-REML was also used to estimate the genetic correlation between RDW and CHD.

To determine whether differences in heritability between the young/old groups was statistically significant we computed the t-statistic using Eq (1) from which a two-tailed p-value is derived.

$$t=\frac{B_{1}-B_{2}}{\sqrt{SE_{1}{}^{2}+SE_{2}{}^{2}}}$$ (1)

### Genome-wide association study

We used data from the May 2015 release of the UK Biobank genetics data, where 152,248 participants were genotyped. Two custom Affymetrix genotyping arrays were used to directly genotype >800,000 genetic variants; the UK BiLEVE array (n = 49,922) and the UK Biobank Axiom array (n = 102,326), which share >95% of genomic markers. Both were enriched for known disease variants and include >600,000 variants specifically chosen to optimize imputation in populations of European descent. Quality control steps performed centrally by UK Biobank [34] included filtering less reliable genotyping results (e.g. setting genotypes to missing in batches where the SNP deviated from Hardy-Weinberg Equilibrium) and identifying poor quality samples (e.g. extreme heterozygosity and/or low call rate) according to Affymetrix recommendations.

Genotype imputation, also performed centrally by UK Biobank [35], was performed on a subset of genotype data, where SNPs with MAF <1% were excluded, leaving 641,018 variants. Phasing and imputation were performed separately, using a custom SHAPEIT2 algorithm to handle large sample size and IMPUTE2 with a combined UK10K and 1000 Genomes Phase 3 reference panel. This resulted in 73,355,667 genetic variations available in the dataset.

We performed a GWAS in 116,666 white/British participants (by self-report & genetic ancestry) with complete genetic data to determine genetic variants associated with RDW. After quality control and filtering (we included autosomal variants with MAF ≥0.1%, missingness <1.5%, imputation quality >0.4 and with Hardy-Weinberg equilibrium (HWE) p>1x10^-6^ within the white/British participants) 16,889,199 imputed genetic variants were available for GWAS analysis: methods described in detail previously [34–36]. We also utilized data directly from the microarrays for variants on the X (n = 19,381) and Y (n = 284) chromosomes, and on the mitochondrial genome (n = 135), which were unavailable in the imputed dataset.

GWAS was performed using BOLT-LMM, a software that uses linear mixed-effect models to determine associations between each variant and the outcome, incorporating genetic relatedness [33]. We provided all directly genotyped SNPs that were present in the imputed data to BOLT-LMM to construct the model. For X, Y and mitochondrial variants we used Plink (v1.9) [37] to determine the associations between genotype and phenotype, with additional adjustment for the first 5 principal components from FlashPCA [38] based on 95,535 independent SNPs (pairwise r^2^<0.1) with MAF >2.5%, missingness <1.5%, and HWE p>1x10^-6^.

The main outcome of the GWAS was RDW residuals from a linear regression model adjusted for age, sex, and assessment center, and were quantile-normalized prior to analysis. Models were adjusted for array type (two different Affymetrix arrays were used, which are >95% identical) at run time. Variants were classed as significant if the p-value for the association with RDW was less than 5x10^-8^.

### Identifying conditionally independent GWAS signals

Many of the identified variants are correlated and may therefore not be independent; we used conditional analysis to determine independent signals by adjusting each variant in a locus for the most significant variant in that locus (loci defined as runs of SNPs separated by <2Mb on a chromosome). This process was repeated until only “conditional SNPs” remained that were significantly associated with RDW independent of one another.

Conditional SNPs were checked for their consistency of association with RDW in two sensitivity analyses: once excluding participants with prevalent anemia (either clinical diagnosis, self-report, or raised hemoglobin), and in a separate analysis excluding participants <60 years of age.

### GWAS-significant SNPs follow-up

The determine the gene-locations and possible functional consequences of the conditionally independent SNPs we submitted them to the UCSC Variant Annotation Integrator (https://genome.ucsc.edu/cgi-bin/hgVai), which combines information from several sources to determine the probable effect of a genetic variant, including on specific genes (for example intronic, missense, splice site, intergenic etc.) and PolyPhen-2 (a tool for predicting the impact of amino acid substitutions on the protein product). We used data from the “Blood eQTL browser” (http://genenetwork.nl/bloodeqtlbrowser) published by Westra *et al*. which reports associations between genetic variants and gene expression in whole blood [25] to determine whether the independent SNPs affect gene expression in human whole blood.

The GWAS catalogue of published variant-trait associations was searched for all SNPs (not just conditional SNPs) associated with RDW to determine which loci had previously been associated with another trait in GWAS analyses (p<5x10^-8^), and which were novel [39]. We used the UCSC `liftOver`tool (https://genome.ucsc.edu/cgi-bin/hgLiftOver) to match the genomic coordinates between the GWAS catalogue (GRCh38) and the UK Biobank genetics data (GRCh37).

We used the software package MAGENTA [13] to determine whether any biological pathways were enriched in the GWAS results, using the pathways database file GO_PANTHER_INGENUITY_KEGG_REACTOME_BIOCARTA. These methods involve adjustment for multiple testing and include information on the expected and observed number of significant genes in each pathway.

### Genetic risk scores

Twenty Genetic Risk Scores (GRS) were computed for each participant based on the number of trait-raising alleles they have for a particular phenotype, such as LDL cholesterol or type-2 diabetes. The derivation of each individual score is described in the **S1 Methods** document. To compute the GRS we used Plink function `scores`[37] which computes the number of trait-raising alleles (0, 1 or 2) in each participant, multiplied (weighted) by the effect size (coefficient or odds ratio) from the previously published study. Each of the 20 GRS computed was tested for its association with RDW using linear regression models, adjusted for age, sex, assessment center, genotype array, and population stratification (using the first 5 principal components (PCs)).

## Supporting information

S1 Table Genetics variants (signals) independently associated with RDW. (XLSX)

S2 Table Variant annotation information for the independent signals associated with RDW. (XLSX)

S3 Table Whole blood eQTL information for the RDW signals. (XLSX)

S4 Table Known associations from the GWAS catalogue for the RDW signals. (XLSX)

S5 Table Pathways significantly enriched in the genetic variants associated with RDW. This analysis using the MAGENTA software included all genetic variants used in the analysis, not just the significant independent signals.
(XLSX)

S6 Table Genetic risk score associations with RDW. (XLSX)

S1 Fig QQ plot of the GWAS of RDW. (PNG)

S1 Methods (DOCX)
