# Supplementary figures and images for: Red blood cell distribution width: Genetic evidence for aging pathways in 116,666 volunteers
Source: PLoS One. 2017 Sep 28;12(9):e0185083. doi: 10.1371/journal.pone.0185083 (PMC5619771; doi:10.1371/journal.pone.0185083)

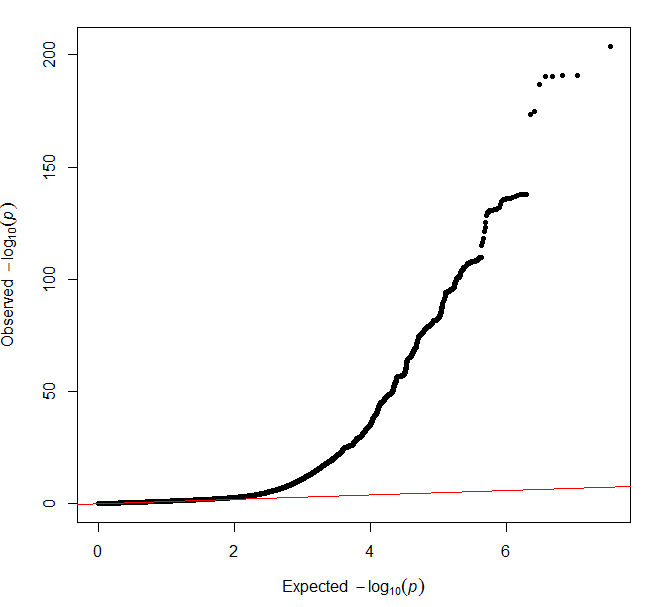

Supplement: S1 Fig — (PNG) [file pone.0185083.s007.png]
