# Supplementary material for: Red blood cell distribution width: Genetic evidence for aging pathways in 116,666 volunteers
Source: PLoS One. 2017 Sep 28;12(9):e0185083. doi: 10.1371/journal.pone.0185083 (PMC5619771; doi:10.1371/journal.pone.0185083)
Supplement: S1 Methods — (DOCX) [file pone.0185083.s008.docx]

UK Biobank RDW genetics paper

Pilling et al. 2017

Supplementary methods document

Contents

[UK Biobank RDW genetics paper 1](#_Toc488064233)

[Selection of variants for GRS 2](#_Toc488064234)

[Body Mass Index 2](#_Toc488064235)

[Height 2](#_Toc488064236)

[Coronary Artery Disease 2](#_Toc488064237)

[Lipids: LDL, HDL and TG 2](#_Toc488064238)

[Coronary Artery Disease – independent of lipids 2](#_Toc488064239)

[Systolic Blood Pressure 3](#_Toc488064240)

[Stroke 3](#_Toc488064241)

[Alzheimer’s Disease 3](#_Toc488064242)

[Breast Cancer 3](#_Toc488064243)

[Prostate Cancer 3](#_Toc488064244)

[Colorectal Cancer 3](#_Toc488064245)

[Inflammatory Bowel Disease 4](#_Toc488064246)

[Forced Vital Capacity 4](#_Toc488064247)

[Telomere Length 4](#_Toc488064248)

[Type-1 diabetes 4](#_Toc488064249)

[Type-2 diabetes 5](#_Toc488064250)

[References 6](#_Toc488064251)

# Selection of variants for GRS

## Body Mass Index

We selected 69 of 76 common genetic variants associated with BMI at genome wide significance in the GIANT consortium studies of up to 339,224 individuals [1]. We limited the BMI SNPs to those that were associated with BMI in the analysis of all European ancestry individuals and did not include those that only reached genome-wide levels of statistical confidence in one-sex only, or one-strata only. Variants were also excluded if classified as a secondary signal within a locus. [Three SNPs were excluded due to potential pleiotropy (rs11030104, rs13107325, rs3888190)], 3 SNPs were not in Hardy-Weinberg Equilibrium (HWE) (rs17001654, rs2075650, rs9925964) and one SNP was unavailable (rs2033529).

## Height

Of the 423 height-associated loci identified by Wood *et al.* in a meta-analysis of 253,288 individuals of European ancestry [2], we excluded 18 which were not independent (R^2^>0.1) of another signal at the same locus. Of the remaining 405 there were 3 that significantly deviated from HWE (p<1x10^-6^), leaving 402 genetic variants used to generate the GRS.

## Coronary Artery Disease

Of 44 genetic variants associated (p<5x10^-8^) with Coronary Artery Disease (CAD) in the recent meta-analysis of up to 185,000 cases and controls [3] one was excluded based on R^2^>0.1 with another SNP (<1Mb distance) in the GRS, and one other was included as it was not available in the data; leaving 42 eligible SNPs to include.

## Lipids: LDL, HDL and TG

The most recent Global Lipids Consortium meta-analysis [4] (n=188,577) reported the following numbers of variants associated (p<5x10^-8^) with: LDL (55), HDL (67), and TG (37). Three LDL SNPs were not in HWE (rs1800562, rs2902940, rs1250229) so were excluded. No further exclusions were required. Therefore 52 LDL SNPs, 67 HDL SNPs and 37 TG SNPs were included in the respective GRS’.

## Coronary Artery Disease – independent of lipids

Of the 42 SNPs included in the CAD GRS we excluded 9 where the SNP (or an exact proxy) was present in the LDL meta-analysis and associated with LDL p<0.05, an additional 4 SNPs that were associated p<0.05 with HDL, and 6 SNPs for which no proxies in the lipid GWAS could be identified, to create a “CAD no lipids” GRS including 23 SNPs associated with CAD but not LDL, HDL or TG.

## Systolic Blood Pressure

We generated GRS for systolic blood pressure (SBP) only, due to the substantial overlap between the SNPs identified for SBP and diastolic blood pressure, based on the results of a meta-analysis on up to 200,000 participants of European ancestry [5]. All 26 SNPs for SBP were present in the UK BioBank imputation data with quality >0.95, and all HWE p-values >1x10^-6^.

## Stroke

In 2014 a GWAS of 17,970 ischemic stroke cases reported 4 loci associated with stroke (rs17696736, rs2023938, rs12646447, rs10744777) [6]. All four were present in the imputed Biobank genetics data with quality >0.95 and HWE p-values >1x10^-6^.

## Alzheimer’s Disease

Results from a meta-analysis including 4,018 Alzheimer’s Disease (AD) cases showed 7 loci significantly associated with late-onset AD [7]. All 7 were used to create the GRS in this study (imputation quality =1 for all SNPs, HWE p>1x10^-6^).

## Breast Cancer

The most recent breast cancer GWAS meta-analysis found 41 new loci, bringing the total to 66 [8]. One SNP (rs614367) was excluded due to HWE p<1x10^-6^, leaving 65 for the GRS.

## Prostate Cancer

The most recent GWAS meta-analysis identified 23 new loci for prostate cancer (PC), bringing the total to 87 [9]. We used the results from participants on European descent (35,093 cases, 34,599 controls) to generate the risk score. All SNPs have imputation quality >0.95, however 2 SNPs (rs1775148 and rs1983891) were excluded due to HWE p<1x10^-6^, meaning 85 SNPs were included in the GRS.

## Colorectal Cancer

Schumacher *et al*. replicated 41 previously reported associations and identified 6 new loci for colorectal cancer, which were either genome-wide significant in the discovery set or when meta-analyzed with an independent cohort of Asian ancestry [10]. We used the odds ratios from the analysis of participants of European ancestry only. Two SNPs were excluded due to missing data (rs35509282 and rs12241008). Nine more SNPs were excluded due to correlation (R^2^>0.1) with another SNP at the same locus; the SNP with the lowest p-value at each locus was retained. We therefore included 36 SNPs in the GRS.

## Inflammatory Bowel Disease

Inflammatory Bowel Disease (IBD) is commonly defined as presence of either Crohn’s Disease (CD) or Ulcerative Colitis (UC), and a recent GWAS meta-analysis found significant overlap between CD and UC, but also some genetic divergence [11]. In total 232 unique SNPs are reported with either CD, UC or IBD in the 86,640 individuals of European descent in the study, 142 with CD, 89 with UC, and 159 with IBD. We generated three GRS based on these lists: for IBD 3 SNPs were excluded due to HWE p<1x10^-6^ (rs4703855, rs224090, rs17622378), for CD the same 3 SNPs were excluded, and for UC 2 SNPs were excluded due to HWE p<1x10^-6^ (rs17622378 and rs17771967 – the latter is unique to the UC list of significantly associated SNPs). Therefore the following number of SNPs were included: 156 for IBC, 139 for CD and 87 for UC.

## Forced Vital Capacity

In an analysis of 85,170 participants for Forced Vital Capacity (FVC) 6 loci were significant in the combined meta-analysis [12]. All were imputed in UK BioBank with high quality (>0.95) and with HWE p>1x10^-6^. Therefore all 6 SNPs were included in the FVC GRS.

## Telomere Length

Seven loci were identified to be associated with Telomere Length (TL) in a recent GWAS meta-analysis [13]. All 7 were imputed with high quality and no significant deviation from HWE was observed, therefore all 7 contributed to the GRS. Greater values of TL GRS equate to longer telomeres.

## Type-1 diabetes

In total 29 SNPs were used to create the GRS for Type-1 Diabetes (T1D), using the methods as described in [14]. Two SNPs (rs2187668, rs7454108) were used to determine an individual’s haplotype combination (DR3/DR4-DQ8) with each haplotype combination having an associated effect size. 27 out of 30 non-HLA SNPs were utilized in addition to the HLA haplotype-tagging SNPs (3 were excluded due to HWE p<5x10^-6^).

## Type-2 diabetes

Of 65 loci confirmed to be associated with Type-2 Diabetes (T2D) in a recent GWAS meta-analysis (34,840 cases) [15] 10 were excluded due to non-specific effects (e.g. primary effect of rs12970134 is on BMI, therefore excluded here) and one was substituted for a proxy (rs11651052 substituted for rs4430796). Therefore 55 SNPs are included in the GRS.

# References

1. Locke AE, Kahali B, Berndt SI, Justice AE, Pers TH, Day FR, et al. Genetic studies of body mass index yield new insights for obesity biology. Nature [Internet]. 2015;518:197–206. Available from: http://www.ncbi.nlm.nih.gov/pubmed/25673413

2. Wood AR, Esko T, Yang J, Vedantam S, Pers TH, Gustafsson S, Chu AY, Estrada K, Luan J, Kutalik Z, Amin N, Buchkovich ML, Croteau-Chonka DC, Day FR, Duan Y, Fall T, Fehrmann R, Ferreira T, Jackson AU, Karjalainen J, Lo KS, Locke AE, Mägi R, Mihailov E, Por FT. Defining the role of common variation in the genomic and biological architecture of adult human height. Nat Genet. 2014;46:1173–86.

3. Nikpay M, Goel A, Won H-H, Hall LM, Willenborg C, Kanoni S, et al. A comprehensive 1000 Genomes–based genome-wide association meta-analysis of coronary artery disease. Nat. Genet. [Internet]. 2015;47:1121–30. Available from: http://www.nature.com/doifinder/10.1038/ng.3396

4. Global Lipids Genetics Consortium, Willer CJ, Schmidt EM, Sengupta S, Peloso GM, Gustafsson S, et al. Discovery and refinement of loci associated with lipid levels. Nat. Genet. [Internet]. 2013;45:1274–83. Available from: http://www.nature.com/doifinder/10.1038/ng.2797

5. Ehret GB, Munroe PB, Rice KM, Bochud M, Johnson AD, Chasman DI, et al. Genetic variants in novel pathways influence blood pressure and cardiovascular disease risk. Nature [Internet]. Nature Publishing Group; 2011 [cited 2011 Nov 21];478:103–9. Available from: http://www.ncbi.nlm.nih.gov/pubmed/21909115

6. Kilarski LL, Achterberg S, Devan WJ, Traylor M, Malik R, Lindgren A, et al. Meta-analysis in more than 17,900 cases of ischemic stroke reveals a novel association at 12q24.12. Neurology [Internet]. 2014;83:678–85. Available from: http://www.ncbi.nlm.nih.gov/pubmed/25031287

7. Kamboh MI, Demirci FY, Wang X, Minster RL, Carrasquillo MM, Pankratz VS, et al. Genome-wide association study of Alzheimer’s disease. Transl. Psychiatry. 2012;1–7.

8. Michailidou K, Hall P, Gonzalez-Neira A, Ghoussaini M, Dennis J, Milne RL, et al. Large-scale genotyping identifies 41 new loci associated with breast cancer risk. Nat. Genet. [Internet]. 2013;45:353–61, 361-2. Available from: http://www.ncbi.nlm.nih.gov/pubmed/23535729

9. Al Olama AA, Kote-Jarai Z, Berndt SI, Conti D V, Schumacher F, Han Y, et al. A meta-analysis of 87,040 individuals identifies 23 new susceptibility loci for prostate cancer. Nat. Genet. [Internet]. 2014;46:1103–9. Available from: http://www.pubmedcentral.nih.gov/articlerender.fcgi?artid=4383163&tool=pmcentrez&rendertype=abstract

10. Schumacher FR, Schmit SL, Jiao S, Edlund CK, Wang H, Zhang B, et al. Genome-wide association study of colorectal cancer identifies six new susceptibility loci. Nat. Commun. [Internet]. 2015;6:7138. Available from: http://www.nature.com/doifinder/10.1038/ncomms8138

11. Liu JZ, van Sommeren S, Huang H, Ng SC, Alberts R, Takahashi A, et al. Association analyses identify 38 susceptibility loci for inflammatory bowel disease and highlight shared genetic risk across populations. Nat. Genet. [Internet]. 2015;advance on. Available from: http://dx.doi.org/10.1038/ng.3359

12. Loth DW, Artigas MS, Gharib SA, Wain L V, Franceschini N, Koch B, et al. Genome-wide association analysis identifies six new loci associated with forced vital capacity. Nat. Genet. [Internet]. 2014;46:669–77. Available from: http://www.nature.com/doifinder/10.1038/ng.3011

13. Codd V, Nelson CP, Albrecht E, Mangino M, Deelen J, Buxton JL, et al. Identification of seven loci affecting mean telomere length and their association with disease. Nat. Genet. [Internet]. 2013 [cited 2013 Mar 27];45:422–7. Available from: http://www.nature.com/doifinder/10.1038/ng.2528

14. Oram RA, Patel K, Hill A, Shields B, McDonald TJ, Jones A, et al. A Type 1 Diabetes Genetic Risk Score Can Aid Discrimination Between Type 1 and Type 2 Diabetes in Young Adults. Diabetes Care [Internet]. 2015;1–8. Available from: http://care.diabetesjournals.org/cgi/doi/10.2337/dc15-1111

15. Morris AP, Voight BF, Teslovich TM, Ferreira T, Segrè A V, Steinthorsdottir V, et al. Large-scale association analysis provides insights into the genetic architecture and pathophysiology of type 2 diabetes. Nat. Genet. [Internet]. 2012;44:981–90. Available from: http://www.ncbi.nlm.nih.gov/pubmed/22885922
